# Perspectives on skeletal muscle stem cells

**DOI:** 10.1038/s41467-020-20760-6

**Published:** 2021-01-29

**Authors:** F. Relaix, M. Bencze, M. J. Borok, A. Der Vartanian, F. Gattazzo, D. Mademtzoglou, S. Perez-Diaz, A. Prola, P. C. Reyes-Fernandez, A. Rotini, Taglietti

**Affiliations:** 1 Univ Paris Est Creteil, INSERM, IMRB, 94010 Creteil, France; 2 EnvA, IMRB, 94700 Maisons-Alfort, France; 3 EFS, IMRB, 94010 Creteil, France; 4 AP-HP, Hopital Mondor, Service d’histologie, 94010 Creteil, France

**Keywords:** Muscle stem cells, Skeletal muscle

## Abstract

Skeletal muscle has remarkable regeneration capabilities, mainly due to its resident muscle stem cells (MuSCs). In this review, we introduce recently developed technologies and the mechanistic insights they provide to the understanding of MuSC biology, including the re-definition of quiescence and G_alert_ states. Additionally, we present recent studies that link MuSC function with cellular heterogeneity, highlighting the complex regulation of self-renewal in regeneration, muscle disorders and aging. Finally, we discuss MuSC metabolism and its role, as well as the multifaceted regulation of MuSCs by their niche. The presented conceptual advances in the MuSC field impact on our general understanding of stem cells and their therapeutic use in regenerative medicine.

## Abstract (web summary)

Skeletal muscle has a remarkable regenerative capacity, which can largely be attributed to resident muscle stem cells (MuSCs). Here, the authors review the molecular mechanisms regulating MuSC quiescence, activation and proliferation, how these processes are regulated by the stem cell niche, and the role of MuSCs in neuromuscular diseases.

## Introduction

Representing 30–40% of our body mass, skeletal muscle is a highly organized tissue made up of a large number of syncytial cells, known as myofibers, which are formed by the fusion of myogenic progenitor cells. Despite the post-mitotic nature of its myofibers, skeletal muscle has a robust regenerative capacity in response to injury. This relies on resident muscle stem cells (MuSCs), also called “satellite cells” because of their unique anatomical position at the periphery of the myofibers. MuSCs typically exist in a quiescent state but may enter the cell cycle following injury in order to regenerate the skeletal muscle tissue and replenish the stem cell pool for future needs. Several transcription factors have been identified as markers and key regulators of the quiescent state as well as of activation and progression to the myogenic lineage. Among them, the paired homeobox factors PAX3 and PAX7 as well as the so-called Myogenic Regulatory Factors – MRFs (MYF5, MYOD, MYOGENIN, MRF4) stand out for their unique and important roles in muscle formation, specification, homeostasis, and repair (for more details the reader may refer to ref. ^1,2^). PAX7 is commonly used as a marker of MuSCs, and a subset of them co-expresses PAX3 in adult muscle^3,4^. The MRFs regulate the progression of MuSCs towards myogenic determination, differentiation, and fusion to form multinucleated myofibers^2^.

The renewal of the MuSC cellular compartment requires a tightly regulated balance between quiescence and activation that is associated with many transcriptional changes in MuSCs. Activation is accompanied by metabolic reprogramming, reinforcing the evidence of a strict interplay between MuSC function and metabolic status. Moreover, recent studies show that MuSCs are a heterogeneous stem cell population, with different abilities to support tissue regeneration. The dynamic changes in MuSC behavior are regulated by the microenvironment and by distinct tissue resident cells of the niche that provide molecular cues to regulate MuSC fate. Here, we review novel findings that have challenged our knowledge of MuSC biology, discussing the molecular mechanisms regulating MuSC quiescence and activation states and heterogeneity. Moreover, we describe the latest advances that enhance our understanding of how MuSC metabolism adapts to quiescence and differentiation, and the role of the microenvironmental niche in regulating MuSC behavior and function. Finally, we present new insights into the pathological conditions associated with MuSC dysfunction, such as muscular dystrophies and aging, showing how the deregulation of MuSCs can lead to an exacerbation of pathology.

## State of the art

### MuSC quiescence and activation

#### Capturing the quiescence state

The ability of MuSCs to drive robust tissue regeneration is based on their ability to exit from their steady-state quiescent state and pass to an activated state following stimuli encountered notably in traumatic or pathological conditions^2^. Initially, the quiescent and activated states were defined at the molecular level by PAX7 expression and by rapid MRF (e.g. MYF5, MYOD) upregulation, respectively. Following activation, MuSCs start to proliferate; the majority differentiates for muscle repair, while a subpopulation of activated/proliferating MuSCs will replenish the quiescent pool.

However, recent technical advances have revealed a higher level of complexity in the molecular signatures of quiescence and activation. Four groups have independently developed protocols to capture a dormant gradient state of MuSCs by in situ fixation^5,6^, by single-cell sequencing^7^ or by isolation of quiescent cells via TU-tagging^8^. These studies showed that during mechanical and enzymatic tissue dissociation MuSCs undergo rapid changes in transcription and histone modifications^5–8^ and they provide novel experimental approaches to isolate MuSCs in their native state, and to analyze both quiescence and early activation mechanisms. The early response of MuSCs to the disruption of their niche includes increased expression of AP-1 members such as Fos and Jun, rapid downregulation of Hox genes, of genes encoding zinc finger proteins or metabolism enzymes, and of Notch signaling^5–8^.

#### Regulation of quiescent MuSCs

**Fig. 1:**
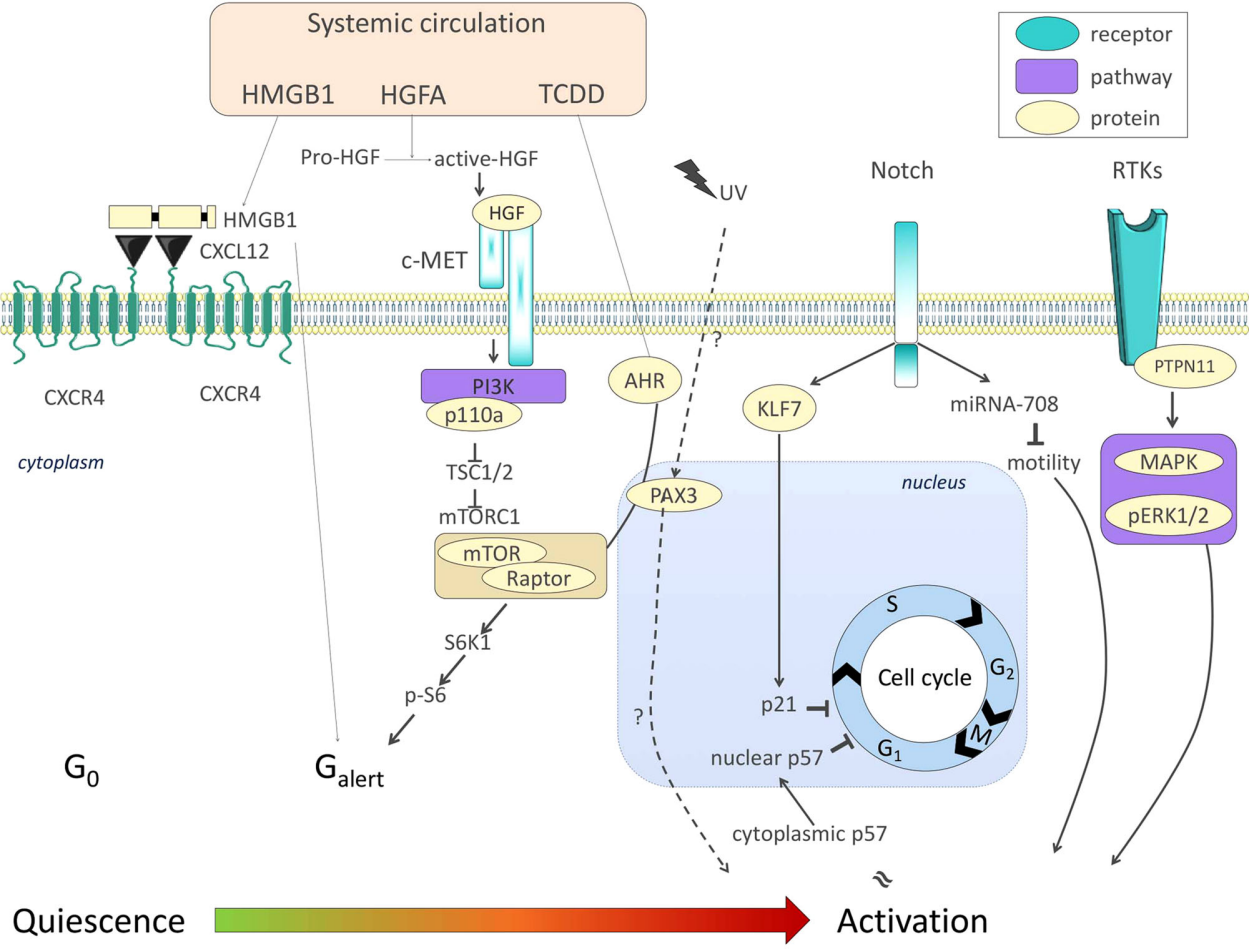
Signaling pathways involved in MuSC transition from quiescence to activation. Systemic signals such as HMGB1, HGFA, and the environmental pollutant TCDD (commonly known as dioxin) promote G_alert_ features in MuSCs through mTORC1 signaling. The Notch signaling pathway, critical for the maintenance of the quiescent state of MuSCs, was recently described to block proliferation and activation through KLF7-p21 and miRNA-708-Notch-migration axes, respectively. Finally, PTPN11/MAPK signals and cytosolic p57 are compatible with proliferation and activation of MuSCs. Color code: blue, receptor; beige, protein; purple, pathway.

How quiescence is maintained is not fully understood, but Notch signaling plays a key role. Notch is active in quiescent MuSCs and interference with canonical Notch signaling results in depletion of MuSCs through spontaneous differentiation^9,10^. More recently, Notch signaling was found to induce the transcription of miR-708 (Fig. 1), which impedes MuSC proliferation and motility^11^. KLF7 is an additional factor that was placed downstream of Notch and was found to be necessary for maintaining MuSC quiescence. Knockdown and overexpression experiments showed that KLF7 limited MuSC cell cycle entry through upregulation of the Cyclin-Dependent Kinase Inhibitor (CDKI) p21 (Fig. 1) but did not affect differentiation^12^. In the same CDKI family as p21, p57 was found to migrate from the cytoplasm to the nucleus to promote cell cycle exit of activated MuSC-derived myoblasts^13^ (Fig. 1). Both studies demonstrated CDKI effects on proliferation but not differentiation, which are frequently concomitantly de-regulated.

#### Regulation of MuSC proliferation

In parallel with studies focusing on the establishment and maintenance of quiescence, recent work explored factors that promote MuSC proliferation, following their activation. MuSC-specific deletion or chemical inhibition of the tyrosine phosphatase PTPN11 negatively affects MuSC cycling status and expansion via alteration in MAPK signaling, causing a reduction of the stem cell pool and impaired tissue repair post injury^14^. p110a, a catalytic subunit of phosphatidylinositol 3-kinase (PI3K) has also been implicated in the control of quiescence exit. Constitutively active p110a promoted spontaneous cell cycle entry, differentiation, and fusion with underlying fibers, while MuSC-specific loss of p110a had the opposite effect. p110a loss of function phenotypes were partially rescued by concomitant MuSC-specific deletion of TSC1, a specific repressor of mTORC1^15^ (Fig. 1). Of note, mTORC1 deletion in the myogenic lineage led to perinatal death, while adult MuSC-specific mTORC1 deletion after injury significantly impaired muscle regeneration^16^. These findings are of particular interest, as mTORC1 has been involved in the quiescence-to-activation transition of MuSCs (see below).

#### Intermediate states between quiescence and activation

The dogma of MuSCs existing in either quiescent or activated state was challenged when an intermediate status was discovered in 2014. Rodgers et al.^17^ described an “alerted” state (G_alert_) that was clearly similar to, but distinct from quiescence, lying between the G_0_/quiescent and G_1_/cycling states (Fig. 1). G_alert_ MuSCs are slightly bigger, are primed for the first division, display increased mitochondrial activity and show higher regenerative capacity^17^. The Rando group first reported this state in limb muscle contralateral to the site of muscle injury, indicating the existence of a systemic signal priming MuSCs to be in the G_alert_ state and they demonstrated that mTORC1 signaling was sufficient to provoke this response^17^. More recently, the same group showed that tissue damage activates the Hepatocyte Growth Factor Activator (HGFA), which in turn binds to c-Met and activates mTORC1 through PI3K^18^ (Fig. 1). The fracture-released alarmin high mobility group box 1 (HMGB1), also supports the idea of a systemic signal placing MuSCs into G_alert_, in this case through CXCL12-CXCR4 signaling^19^ (Fig. 1). As both HGFA and HMGB1 activate stem cells and progenitors outside the muscle lineage, these studies suggest that circulating factors contribute to improved tissue repair and could suggest conceptually different treatment strategies for regenerative medicine.

Apart from priming MuSC activation and tissue repair, G_alert_ was shown to ensure MuSC stemness in the absence of functional impairment in a MuSC subset marked by PAX3. Indeed, Der Vartanian and colleagues^4^ showed that following environmental stress (Dioxin/TCDD exposure) the PAX3+ MuSC subpopulation is blocked in mTORC1-dependent G_alert_ (Fig. 1), showing enhanced survival. In contrast, TCDD-exposed PAX3- MuSCs were lost through atypical activation, and sporadic differentiation, which was dependent on the AHR (Aryl hydrocarbon receptor) pathway of xenobiotic metabolism (Fig. 1)^4^. Similarly, Scaramozza et al.^20^ found that a PAX3-enriched MuSC subpopulation extensively contributed to muscle repair upon radiation stress, in contrast to their PAX3- counterparts. These data imply that the G_alert_ state protected quiescent cells from aberrant proliferation and differentiation under environmental stress, in the same way that quiescence protects cells from proliferation-induced DNA damage^20^.

#### Regulation of activated MuSCs & asymmetric divisions

Once entering the cell cycle, MuSCs display different potential for proliferation, self-renewal, and differentiation after cell division which is linked to cell polarity that is in turn influenced by spatially and temporally defined signals^21^. Normally after asymmetric stem cell division one daughter cell commits to differentiation and the other returns to quiescence, with the latter process being called self-renewal. The precise spatio-temporal mechanism of asymmetric cell division remains unclear since: i) intravital imaging revealed that divisions and migration following muscle injury are oriented along the ghost fibers, which are fiber remnants that act as scaffolds to guide MuSC divisions after injury^22^, ii) yet, daughter cells traced with a *Myf5-Cre* lineage reporter are primed for myogenic differentiation while *Myf5-Cre* negative cells self-renew to maintain the stem cell pool^23^. The coactivator associated arginine methyltransferase 1 (CARM1) is also involved in asymmetric MuSC divisions by methylating PAX7, which in turn activates MYF5 transcription^24^. This activation is prevented by CARM1 phosphorylation. Additional studies have established that the receptor CDO-^25^ and TNFα-activated p38α/β regulate MuSC activation and the ability to undergo asymmetric division and differentiation^26,27^. This regulation is mediated by p38α/β-PAR complex asymmetric localization in only one daughter cell^26^, as well as p38-mediated regulation of PAX7 (via Polycomb Complex)^27^ and cell cycle^28^.

**Fig. 2:**
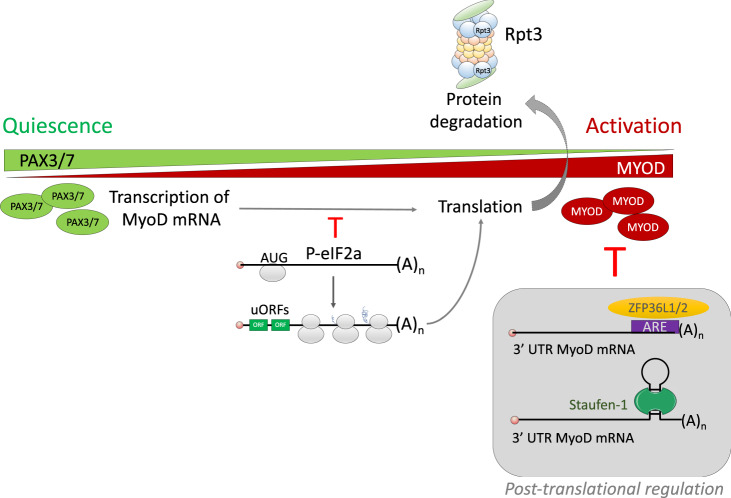
Transcriptional regulation controls MuSC quiescence. While MuSC quiescence is primarily associated with PAX3/7 expression, MYF5 and MYOD are hallmarks of MuSC activation and myogenic commitment. *Myf5/MyoD* transcripts are expressed in quiescent MuSCs, but they are sequestered in messenger ribonucleoprotein (mRNP) granules. Such granules contain RNA-binding proteins like ZFP36 and Staufen-1, which act in granule assembly and mRNA sequestration, respectively. Dissociation from the mRNP granules and translation initiation is mediated by phosphorylation of the eIF2a protein. Color code of quiescence-to-activation trajectory: green, quiescence; red, activation.

The microRNA (miR) pathway is also essential to prevent the translation of transcripts involved in MuSC activation. Such transcripts include *MYF5*^29^ and *DEK*, which encode proteins localized to the daughter cell that will undergo differentiation following asymmetric division of MuSCs^30^. miRNAs targeting these transcripts ensure that quiescent MuSCs do not activate the myogenic program or enter the cell cycle, respectively. Both miRNA-31 and *MYF5* were found in structures called the messenger ribonucleoprotein granules (mRNP), which can be dissociated to release MRF mRNA during activation^29^. This dissociation requires factors such as the eukaryotic initiation factor 2a (eIF2a) to maintain MuSCs in quiescence^31^ (Fig. 2). The mRNP also contains RNA-binding proteins such as ZFP36L1/2 and STAUFEN-1, which act in granule assembly and mRNA sequestration to maintain quiescence^32–34^. RNA granules maintain quiescence by negatively regulating the translation of MYOD through proteins binding to the 3′UTR of MYOD and stimulating mRNA degradation^34^ (Fig. 2).

MuSC quiescence and activation are also regulated by proteasome-mediated degradation. Specifically, MuSC-specific deletion of RPT3, an essential subunit of the 26 S proteasome, led to progressive loss of MuSCs and lack of tissue repair following muscle injury^35^. Indeed, these proteins influence MuSC activation and number, muscle mass and regenerative capacity following muscle injury.

### MuSC heterogeneity

The complex regulation of quiescence and activation discussed above is associated with substantial biochemical and functional diversity linked to MuSC heterogeneity. Such heterogeneity has been described at several levels^21^, including transcription factor and surface marker expression, clonal capacity, transplantation efficiency, metabolism, age-dependent myogenic potential, and recently functional response to environmental stress^4,20^.

#### Heterogenic expression of key MuSC regulatory factors

The myogenic factor PAX7 has been commonly used as a marker of quiescent MuSCs, and new PAX7 mouse reporter lines are being generated to follow the dynamic fate of MuSCs in vivo^36^. Interestingly, heterogeneity in PAX7 expression was recently observed in human MuSCs as a consequence of exposure to a simulated microgravity (10^-3^ G) condition. Microgravity was found to decrease PAX7 expression through an ERK-mediated pathway^37^. Together with Pax7, a subset of MuSCs co-expresses the transcription factor PAX3 in adult muscle^3,4^. As mentioned above, MuSCs adopt different responses to environmental stress according to PAX3 expression, with the PAX3+ compartment being resistant to dioxin^4^ and irradiation^20^. How PAX3 is transcriptionally differentially regulated in satellite cells remains an open question. Recent work from the Rando lab has added another layer of complexity by demonstrating that PAX3 levels govern the variation of MuSC activation in distinct muscles. PAX3 expression is regulated by alternative polyadenylation of its transcript, via the small nucleolar RNA U1, leading to distinct accessibility by miR-206^38^.

Furthermore, new powerful technologies, such as in vivo live imaging, RNA-seq and single-cell deep sequencing (scRNA-seq), are valuable tools to elucidate cell heterogeneity during homeostasis, regeneration, and aging. For instance, scRNA-seq confirmed the heterogeneous level of PAX7 expression inside the MuSC population^39^. scRNA-seq performed on 21 single freshly isolated MuSCs revealed that protein ubiquitination, post-translational modifications, and metabolic pathways were major contributors to MuSC heterogeneity^40^. Recently, Dell’Orso et al.^41^ extended the scRNA-seq analysis (3081 freshly-isolated MuSCs), confirming the transcriptional heterogeneity of the MuSC pool. Based on t-distributed stochastic neighbor embedding (t-SNE) analysis, MuSCs were grouped into two different clusters: one enriched for genes associated with quiescence *(HES1*, *PAX7*, and the calcitonin receptor-CALCR), and the other containing genes involved in the early state of activation (*MYOD*, *MYF5, RPL24, RPL27*)^41^. Such differences likely reflect differential activation states of the MuSCs. Moreover, MuSCs heterogeneity has also been observed in the regenerating environment^41–43^. Single-cell mass cytometry analysis^42^ conducted on isolated MuSCs after acute injury revealed that MuSCs express a novel combination of surface markers (CD9, CD104) in the transition toward the differentiation program.

The dynamics of the regeneration process have also been observed at single-cell level^44^. Indeed, De Micheli et al.^44^ performed scRNA-seq analysis on TA muscles of adult wild-type mice at 0, 2, 5, and 7 days after injury and found that cycling MuSCs express heterogeneous levels of Syndecan-1/2, a transmembrane heparin sulfate proteoglycan involved in cell proliferation and cell–matrix interaction. This finding suggests that alterations in Syndecan signaling coordinate stage-specific myogenic cell fate regulation.

#### MuSC heterogeneity at different life stages

Finally, heterogeneity has been reported between young and aged MuSCs (for review, see ref. ^45^). Multicolor lineage tracing experiments with *Pax7-CreER*^*TM*^; *R26R*^*Brainbow2.1*^ mice were used to compare the clonal expansion of young and aged MuSCs in vivo^46^. This study demonstrated that the clonal complexity of aged MuSCs is unchanged compared to young MuSCs following injury, but this complexity of aged MuSCs is progressively reduced after multiple injuries^46^. This study sheds light on the behavior of aged MuSCs, which have been previously described to be heterogeneous in proliferation and differentiation potential when compared to their young counterparts (for review, see ref. ^45^).

Taken together, all these new methodologies will uncover the mechanisms regulating MuSC heterogeneity. In the future, this will allow the selective isolation of stem/progenitor cells in a quiescent state or differentiated state that is crucial for stem-cell-based therapy. Nevertheless, heterogeneity is also observed at metabolic levels, as further discussed below.

### MuSC metabolism and regulation of myogenesis

The ability to efficiently adapt metabolism to substrate availability and requirement is known as metabolic flexibility. In the stem cell field, early evidence for metabolic flexibility was provided by the identification of a progressive metabolic shift from glycolysis to oxidative phosphorylation (OXPHOS) during differentiation (for review see ref. ^47^). The substrate preference towards glucose, fatty acids, or amino acids, originally only considered as passive energy providers, has been shown to induce genetic reprogramming through the production of secondary metabolites (for review see ref. ^48^). Similar metabolic patterns were revealed in MuSCs^49,50^, opening new opportunities to control MuSC cell state by targeting metabolic pathways.

#### Quiescent MuSC metabolism

**Fig. 3:**
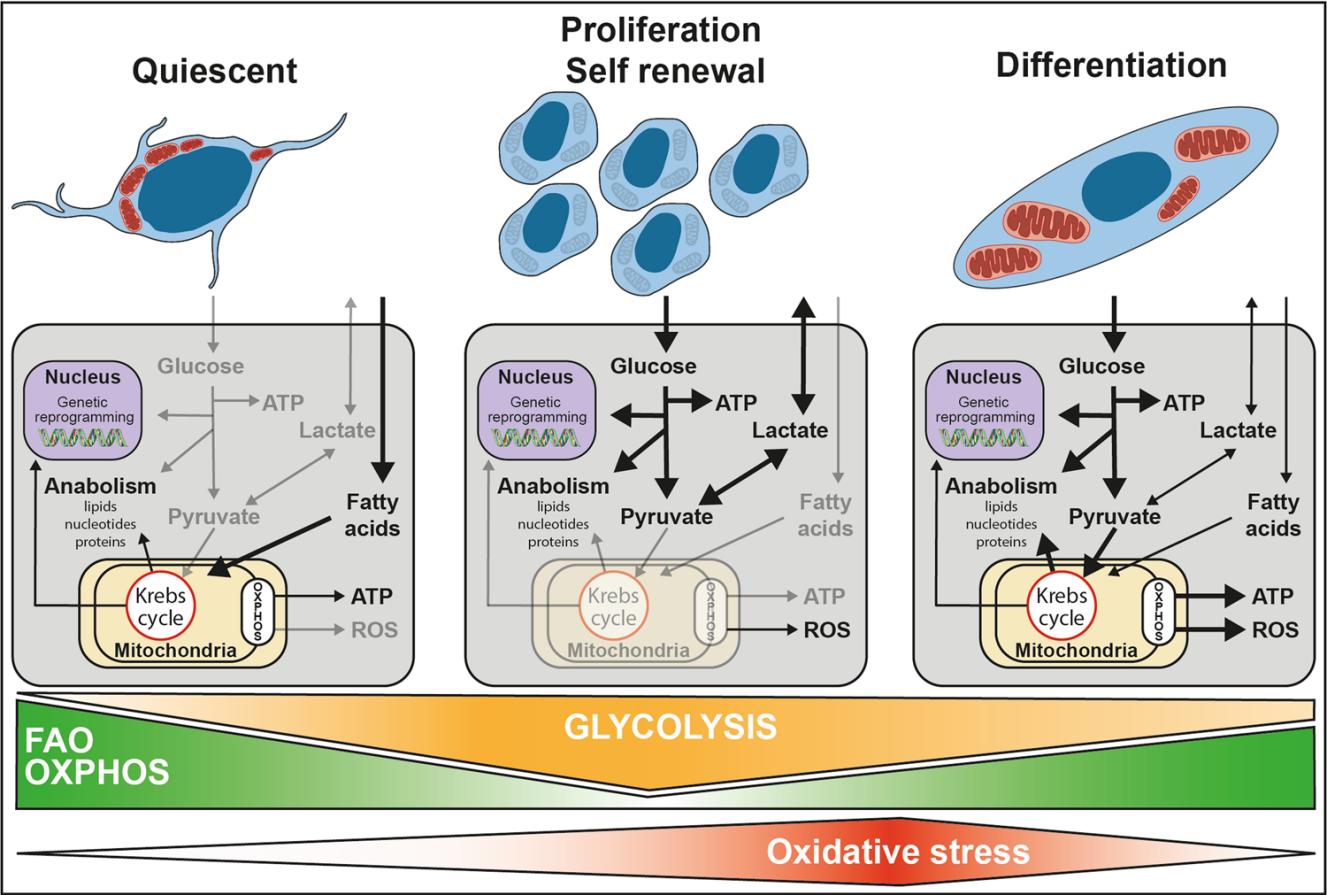
Proposed metabolic pathways regulating quiescence, self-renewal, and differentiation during myogenesis. The low metabolism of quiescent MuSCs is mostly dependent on mitochondrial fatty acid oxidation and oxidative phosphorylation. This promotes epigenetic modification that represses myogenic transcription programs. MuSC activation is associated with a shift toward anaerobic glycolysis. This supports a metabolic environment that allows for rapid biosynthesis, therefore, supporting MuSC growth and proliferation. An increase in mitochondrial respiration precedes MuSC differentiation. This elevated dependence on oxidative phosphorylation triggers a burst of ROS that act as secondary messengers to strengthen differentiation.

Quiescent cells have been described as catabolically inactive and thus likely to preserve their function for years and survive as long as 30 days post-mortem^51^. Data from RNA-seq have provided evidence that quiescent MuSCs are mostly dependent on mitochondrial fatty acid oxidation (FAO) and oxidative phosphorylation (OXPHOS) (Fig. 3). While quiescent MuSCs highly expressed genes involved in fatty acid transport into mitochondria and β-oxidation, the expression of genes involved in glycolysis remained relatively low^5,49^. This dependence on FAO is thought to contribute to the preservation of quiescence at least in part by sustaining high NAD+ levels that induce SIRT1-dependent deacetylation of its target histone H4 lysine 16, in turn repressing myogenic transcription programs^49^. FAO inhibition has been associated with a premature differentiation of MuSCs (Fig. 3), impairing muscle regeneration after injury^52^. In contrast, inducers of FAO such as caloric restriction or increase of NAD+ level improves MuSC function^53,54^. Indeed, increasing NAD+ levels in aged mice favors MuSC quiescence and restores muscle regenerative potential^54^. Caloric restriction enhances regeneration and the capacity of donor MuSCs to efficiently engraft in transplant recipients. However, the effect of caloric restriction on MuSC quiescence remains to be investigated^53^.

Both the predominant FAO and low glycolysis in quiescent MuSCs reduce acetyl-CoA levels, thereby reducing histone and protein acetylation associated with the activation of the myogenic program^55^. Moreover, the prominent FAO is linked to the low metabolic state that is thought to preserve MuSC regenerative potential by minimizing the production of ROS^56^. Accordingly, the knockdown of UCP2, a gene known to favor FAO^57^ triggers a burst of ROS associated with premature differentiation^58^.

However, as MuSC isolation induces the exit of quiescence, the characterization of quiescent MuSC metabolism in situ remains to be investigated.

#### Proliferating MuSC metabolism

The initiation of cell proliferation includes a metabolic shift toward anaerobic glycolysis. The expression of OXPHOS and FAO genes decreased within hours of activation and this was associated with a major increase of anaerobic glycolysis gene expression^5,49,58^ (Fig. 3). Interestingly, inhibition of glycolysis through pharmacological or genetic inhibition of lactate dehydrogenase (LDH) decreased MuSC proliferation and self-renewal, while the overexpression of lactate dehydrogenase A- subunit (LDHA) promoted glycolysis and MuSC expansion. Moreover, the loss of AMPKα1 impaired mitochondrial function, thereby favoring glycolysis and MuSC proliferation^59^.

Aerobic glycolysis that occurs hours after MuSC activation is reminiscent of the Warburg effect observed in cancer cells. The increased glucose uptake is used as a carbon source for anabolic processes to support cell proliferation^60^. This carbon excess is used for the de novo synthesis of nucleotides, lipids, and proteins. Overall, this shift towards anaerobic glycolysis supports a metabolic environment that allows for rapid biosynthesis, therefore supporting MuSC growth and proliferation.

#### Differentiating MuSC metabolism

Differentiation of MuSCs is associated with higher OXPHOS. The expression of genes involved in both, mitochondrial biogenesis and FAO is increased, along with increased respiration^58^ (Fig. 3). This shift toward OXPHOS seems to be necessary as repression of mitochondrial biogenesis by mitochondrial transcription factor A (TFAM) knockdown delayed differentiation^58^.

The role of OXPHOS in MuSC differentiation is mediated by ROS, which are considered as secondary messengers^58^. The activation of MuSCs in response to muscle injury is associated with a burst of ROS production by the respiratory chain in mitochondria and by NADPH oxidases in the cytoplasm^58^. This high level of ROS production is compensated by an increased expression of antioxidant genes such as PITX2 and PITX3, whose deletion induces premature differentiation and loss of regenerative capacity. The role of ROS in the regulation of MuSC differentiation is possibly mediated by the phosphorylation of p38a. Accordingly, inhibition of ROS accumulation by N-acetylcysteine (NAC) treatment or inhibition of p38α phosphorylation by SB203580 prevents MuSC differentiation. Culture of MuSCs in the presence of NAC promotes proliferation, reduces differentiation, and improves their regenerative potential after grafting, opening new possibilities for cell therapy.

To conclude, MuSCs exhibit remarkable metabolic flexibility to adapt to energy needs. The recent demonstration that secondary metabolites can induce major genetic reprogramming provides opportunities to control MuSC state by targeting regulators of MuSC metabolism. It also suggests that a modification of MuSC environment, likely resulting in metabolic alteration, should have important consequences on their function.

### The MuSC niche

MuSCs are surrounded by a variety of cell types^61^ and a complex mesh of extracellular matrix (ECM) proteins, and their interactions are crucial for homeostasis and regeneration. MuSCs receive signals from adjacent myofibers, endothelial cells, pericytes, macrophages, fibro-adipogenic progenitors (FAPs), and likely additional cell types we have little understanding of at this time^61^. These other cell types are known to interact with MuSCs and regulate their quiescence and activation through a number of membrane-bound and secreted factors. The complex microenvironment surrounding the MuSCs and the different support cell populations constitute the niche, which plays an essential role to control MuSC behavior. The dynamic interactions between MuSCs and cells of the niche during muscle regeneration have been recently dissected at the single-cell level using scRNA-seq^44^ and mass cytometry^62^, providing new insights into this fascinating cooperation.

#### Extracellular matrix–basal lamina interaction

**Fig. 4:**
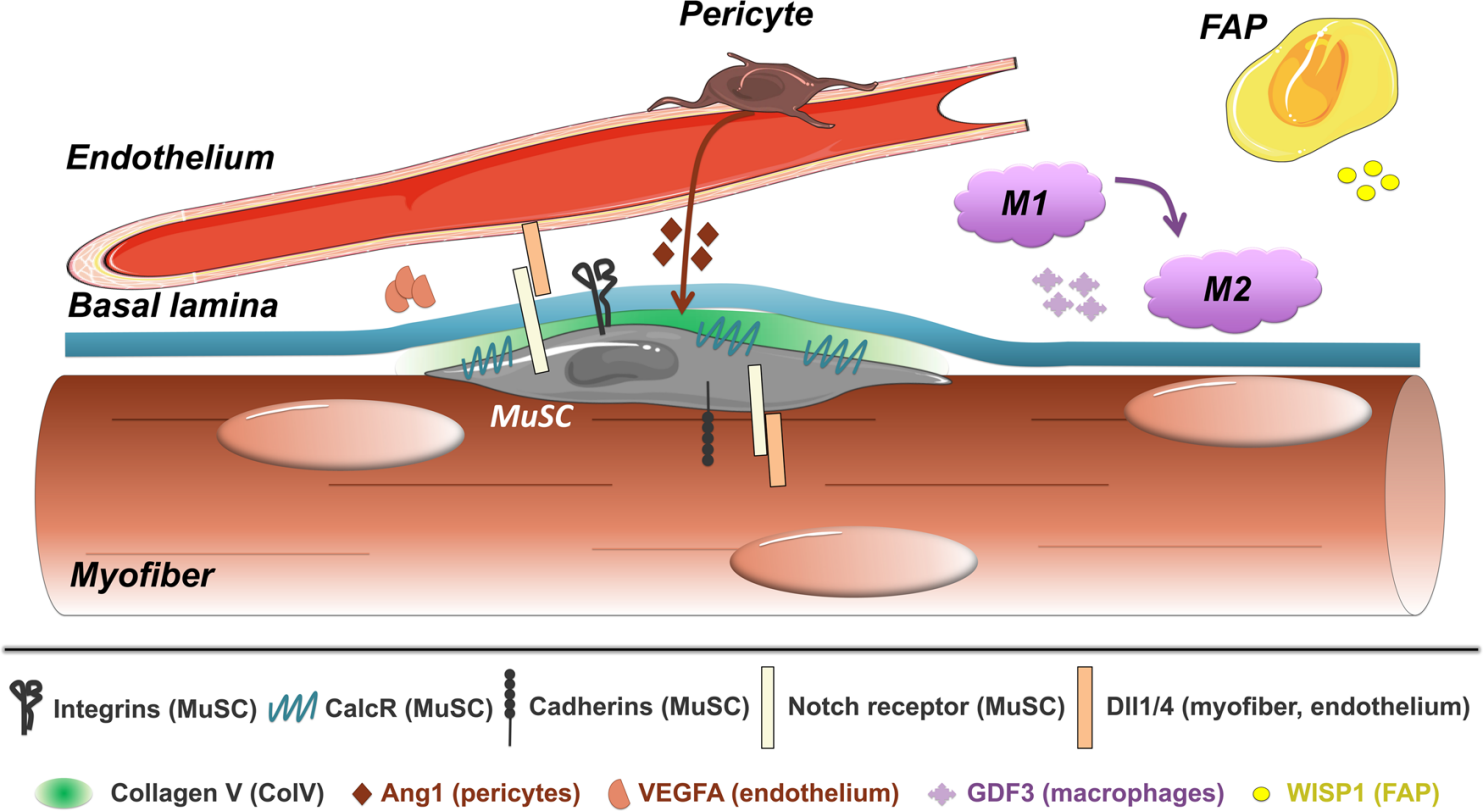
Interaction between the MuSC and its microenvironment. The MuSC attaches to the basal lamina through integrins, which preserve quiescence, though specific integrins can also promote differentiation. Cadherin proteins attach the MuSC to the fiber, which expresses Delta ligands required to maintain a Notch-ON state in the MuSC, necessary for quiescence. Notch stimulates the production of Collagen V, which binds and activates Calcitonin receptor, providing a third axis of quiescence control. Dll4 from the endothelium also sustains MuSC quiescence through Notch signaling, which in turn leads to the production of VEGFA from MuSCs. Angiopoietin-1, secreted by pericytes, further contributes to MuSC quiescence. Upon injury, GDF3 from macrophages and WISP1 from FAPs stimulate differentiation and fusion to ensure efficient regeneration.

The ECM is a dynamic network of macromolecules in the interstitial space with a role in mediating cues that guide stem cell behavior. The MuSCs are located under the basal lamina, a specialized ECM consisting of a mixture of collagen and laminin proteins. Recent work showed that Notch signaling directly activates collagen production by the satellite cells. Culturing MuSCs with Collagen V repressed differentiation and MuSC-specific deletion of Collagen Vα1, a critical subunit of the collagen V structure, led to rapid depletion of the satellite cell pool^63^, recapitulating the phenotype of Notch-deficient MuSCs^9,10^. Finally, the authors demonstrated that Collagen V and MuSC interaction is modulated by the calcitonin receptor (CalcR)^63^ (Fig. 4).

While this work demonstrated a pro-quiescence function for specific collagen proteins, Rayagiri and colleagues^64^ recently found specific laminins involved in MuSC activation. In both injured muscle and cultured muscle fibers, laminin α1 and laminin α5 were increased as MuSCs underwent differentiation. Concordantly, deletion of laminin α1 led to impairment of regeneration after multiple injuries. Finally, the team showed that laminin communicates with MuSCs through integrin α6 (Fig. 4) and that this signal is essential for the maintenance of MuSC polarity and asymmetric cell division^64^.

#### Extracellular matrix–myofiber interaction

The myofiber itself is another source of signals regulating MuSC quiescence and activation. Notch also plays a role in this interaction. Separation of reserve MuSCs from differentiating myofibers showed that NOTCH1 and NOTCH3 were found in MuSCs, while the ligands DLL1 and DLL4 are mainly localized to the myofibers^65^ (Fig. 4). This suggests that in an uninjured muscle the interaction between DLL4 and NOTCH3 contributes to maintaining MuSC quiescence, preventing their activation and differentiation.

Cadherins are known to form critical connections between MuSCs and myofibers, yet analysis of muscle function in knockout mice has been challenging due to the redundancy of certain cadherins and embryonic requirement of others. To circumvent these concerns, Goel and colleagues^66^ generated mice lacking M-cadherin globally, with N-cadherin conditionally deleted in satellite cells. These mice displayed increased numbers of MuSCs and regenerating myofibers without injury from 3 months of age. Cadherin-deficient MuSCs display a partial disruption of the myofiber–MuSC adhesive junction, and while they maintain niche residence and cell polarity, they show properties of MuSCs in a transition state from quiescence to full activation^66^ (Fig. 4). In addition, the recent finding that Wnt4-RhoA axis is a crucial myofiber signal to maintain MuSC quiescence through the repression of YAP^67^ further demonstrates a tight regulation of MuSC functions by the myofibers. These new insights clearly show the importance of myofiber–MuSC interaction to mediate MuSC quiescence and implicate the loss of these signals upon injury as one of the first steps in quiescence exit.

#### Endothelial cells

The close relationship of MuSCs and endothelial cells has been known for some time, and several works have recently further elucidated their molecular interactions^68^. The use of tissue clearing and 3D imaging allowed for the quantitation of MuSC proximity to endothelial cells in situ^69^. Meta-analysis of several published datasets identified VEGFA as a MuSC-derived factor that helps maintain DLL4 expression in endothelial cells (Fig. 4). The VEGF-Notch signaling feedback loop was further confirmed in multiple transgenic models^69^. The communication between endothelial cells and MuSCs was also explored in human cells in vitro, where it was found that co-culture of the cells increases MuSC cell proliferation as well as capillary formation. Several secreted factors enabling coupling between myogenesis and angiogenesis were identified including apelin from endothelial cells, periostin from FAPs, and oncostatin M from anti-inflammatory macrophages^70^. A pro-quiescence and anti-proliferative function for oncostatin M was also demonstrated in mice lacking the receptor specifically in MuSCs^71^. As endothelial-associated cells, pericytes play an important role in regulating the MuSC niche. Angiopoietin secretion by pericytes maintains satellite cell quiescence (Fig. 4), and the ablation of pericytes impaired MuSC behavior, at least partially through pericyte-derived IGF1^72^.

#### Macrophages

Macrophages are a major immune cell population of the muscle and are rapidly recruited after injury. Macrophages are essential for efficient muscle repair: not only orchestrating inflammation and tissue clearance but also contributing to MuSC activation and differentiation^73^.

Du and colleagues^74^ recently showed that macrophages induce MuSC activation through the secretion of ADAMTS1. Adamts1 is a metalloproteinase that suppresses the quiescence regulator NOTCH1 in MuSCs, inducing stem cell activation^74^. GDF3 is another macrophage-secreted factor that is an essential inducer of myoblast fusion and myotube formation^75^ (Fig. 4). Macrophages also secrete glutamine during muscle injury and aging, activating mTOR signaling in MuSCs, promoting their proliferation and differentiation^76^. Due to their relatively transient presence in the muscle, the role of macrophages during regeneration has been challenging to dissect, but there are likely other macrophage-derived signals yet to be discovered.

#### Fibro Adipogenic Progenitors (FAPs)

FAPs have become a cell type of major interest in the muscle field in the last decade, due to their supporting role for the MuSC function during regeneration, and their role in regulating fibrosis and adipocyte infiltration. The identification of Hic1 as a conserved marker of mesenchymal progenitor cells combined with scRNA-seq analysis revealed the existence of different subpopulations of FAPs with distinct cell dynamics in muscle^77^. Further scRNAseq identified subFAPs, characterized by the expression of the surface markers TIE2 and VCAM1, that allow the muscle to adapt to different physiological and pathological stimuli, such as growth and injury^78^. The injury-activated VCAM1+ FAPs display a pro-fibrotic profile, regulate the macrophage-inflammatory response and enable muscle regeneration. The matricellular protein WNT1 Inducible Signaling Pathway Protein 1 (WISP1) was identified as a FAP-derived factor whose expression is increased following injury^79^ (Fig. 4). This response is diminished in aged mice, where the WISP1 secretion by FAPs is blunted, and its loss impairs MuSC functions, controlling asymmetric cell division through the Akt signaling pathway^79^. Finally, ablation of the FAP population leads to defects in immune cell recruitment and MuSC expansion during regeneration^80^.

As our understanding of the MuSC niche increases, the system seems ever more complex. However, this understanding is critical to finding treatments for diseased and aging muscles.

### MuSCs in pathological conditions and therapy

#### MuSCs and muscular diseases

Duchenne muscular dystrophy (DMD) is the prime example of muscle-wasting conditions in which skeletal muscle is subjected to continuous rounds of degeneration/regeneration that impair MuSC function and regenerative capacity. The absence of dystrophin at the sarcolemma is responsible for myonecrosis triggered by mechanical stretch and chronic inflammation. Although TNFα-induced cytotoxicity in dystrophin-deficient mice (mdx) has been shown to induce FAP apoptosis in vitro^81^, there are minimal cleaved-caspase 3-positive nuclei in dystrophin-deficient muscles at the peak of degeneration^82^. These findings suggest the involvement of a caspase-independent cell death mechanism. Morgan and colleagues^82^ demonstrated that TNFα could trigger non-apoptotic cell death in myoblasts in vitro, which requires receptor-interacting protein kinases 1 (RIPK1) and RIPK3 kinase activities. This mode of programmed cell death, called necroptosis, is activated in DMD and mdx muscles. The genetic depletion of RIPK3 in mdx mice resulted in improved myofiber survival and muscle function^82^. Therefore, regulators of the necroptotic pathway could be emerging therapeutic targets for muscular dystrophies. In addition to myofibers, necroptosis has also been recently shown to directly impact MuSC survival during muscle regeneration^83^. Remarkably, necroptotic death of myofiber has recently been directly involved in MuSC proliferation in vivo via the release of Tenascin-C^84^. Whether MuSCs are also susceptible to necroptosis under inflammatory stress in pathological conditions in vivo remains to be established. The long-term effects of necroptosis inhibition on muscle homeostasis in pathology also require to be fully addressed.

One of the major defects in DMD muscle is the presence of fibrosis, and recent work implicated macrophages and FAPs in its development^81^. Juban and colleagues^85^ found that macrophages in mdx mice promoted TGFβ release by FAPs and that fibrosis could be reduced by pharmacologically switching the phenotype of these macrophages. Another promising therapeutic strategy is the reprogramming of fibroblasts into myogenic progenitors via PAX7/MEF2b/MYOD cocktail of transcription factors^86^ or transient activation of MYOD accompanied by exposure to small molecules^87^. These cells expressed MuSC markers and could differentiate into dystrophin-expressing myofibers when grafted into injured muscle.

The field of stem cell therapy has many challenges to overcome. The maintenance of cell quiescence and the preservation of muscle regenerative capacity are critical for the utility of grafted muscle progenitors. While the interaction between dystrophin and the serine-threonine kinase Mark2 is involved in the asymmetric division and self-renewal of MuSCs^88^, Wang et al.^89^ demonstrated that epidermal growth factor (EGF) signals promote asymmetric division via the EGF-receptor and Aurora kinase A (EGFR-Aurka) pathway and improved regenerative muscle capacity and strength in mdx mice^89^. Therefore, extending the capacity of myogenic cells would be of therapeutic interest.

Clonal properties of MuSCs may affect chronically challenged, dystrophic muscles. Nevertheless, Boldrin et al.^90^ described that the number of satellite cells are increased in aged mdx myofibers and retained their engraftment potential^90^. This discrepancy might be due, at least partially, to the relatively poor rate of myonecrosis affecting hindlimb muscles after the necrosis peak occurring at 3–4 weeks of age in the mdx model, and therefore to the relatively modest impairment of mdx MuSCs^91,92^.

An additional adverse effect of the chronic activation of MuSC in muscular dystrophies is the promotion of tumor growth. Indeed, DMD and mdx muscles are more susceptible to develop rhabdomyosarcoma. In a severe DMD mouse model lacking p53 and affected by telomere shortening, the MuSC pool and rhabdomyosarcoma formation were increased^93^. Along the same line, muscle teratomas from adult mice were found to be rich in cells expressing the satellite cell markers α7-integrin and VCAM1. Interestingly, these purified teratoma-derived cells were able to differentiate into functional myogenic progenitors and reconstitute the quiescent MuSC pool following transplantation, displaying remarkable regenerative potential in diseased muscle^94^. Overall, these findings emphasize that fine-tuned protocols and health risk assessments are crucial for the proper manipulation/generation of human MuSCs for clinical applications.

#### MuSC interactions with the aged niche

Aging can affect regulatory cells and signaling pathways in the MuSC niche, disrupting MuSC myogenic potential and regenerative capacity. Therefore, the modulation of MuSC-niche dynamics is an exciting opportunity to restore MuSC function and preserve muscle mass in old and diseased individuals. Age causes alterations in the composition of the niche ECM. For instance, the levels of fibronectin, the preferred niche ECM adhesion substrate for MuSCs, greatly diminish with age^95^. MuSCs can adhere to fibronectin through syndecan-4 and the Wnt receptor Frizzled-7 (SDC4/FZD7) co-receptor complex, which is activated by both WNT7a and fibronectin ligands. Together, Wnt-signals and fibronectin modulate symmetric expansion and self-renewal of MuSCs and are essential for MuSC maintenance during muscle repair^96^. This suggests that the loss of signaling from any of these molecules could lead to impaired muscle regeneration and depletion of the MuSC pool. Moreover, loss of fibronectin from the niche results in impaired integrin-mediated signaling through the p38-MAPK pathway, loss of MuSC number, defective muscle repair, and increased apoptosis^95^. This study demonstrated that alterations in the cells’ adherence capacity are associated with MuSC senescence, and that restoring fibronectin levels in old muscle can rescue MuSC adhesion and muscle regeneration in mice.

Likewise, the restoration of disrupted niche signals has shown positive effects in preserving MuSC activity. As discussed earlier, the age-associated loss of secretion of the matricellular WISP1 factor by FAPs is associated with deterioration in MuSCs. However, the myogenic capacity of MuSCs can be rescued by transplanting young FAPs or systemically restoring the WISP1 signal in aged muscles^79^.

Notch-dependent activation of p53 in MuSCs during muscle regeneration also decreases with age, and this results in impaired self-renewal and a higher incidence of MuSC death via mitotic catastrophe, a mitosis-associated cell death regulated by the Notch-p53 axis^97^. Nevertheless, by stabilizing p53 activity in aged MuSCs, cell death was reduced, and regenerative potential was improved. Similarly, reduced activation of the AMPK/p27^Kip1^ pathway observed in aged MuSCs leads to autophagy impairment and higher susceptibility to apoptotic cell death^98^. Genetic or pharmacological restoration of AMPK or p27^Kip1^ activity effectively suppressed the expression of senescence markers and apoptosis in aged MuSCs^98^, highlighting the AMPK/p27^Kip1^ pathway as a putative target for MuSC rejuvenation.

Age-associated changes can also modify the MuSC transcriptome, inducing functional and metabolic defects that disrupt muscle homeostasis. Solanas et al.^99^ analyzed the effects of aging on the diurnal functional rhythms of skeletal MuSCs in mice. They found that while aged MuSCs retain a rhythmic circadian machinery, their oscillating transcriptome is reprogrammed, switching from homeostasis-related to tissue-specific stress genes, mainly associated with DNA damage and decreased autophagy activity^99^. Interestingly, long-term caloric restriction in mice showed a protective effect against the age-dependent reprogramming of MuSCs, resulting in increased MuSC number and improved stem cell function. Although exciting, these findings are dependent on the time of intervention, and are genetic background- and gender-specific^100^.

## Conclusions and future directions

Skeletal muscle is the most abundant tissue in the human body, required for movement but also playing key physiological roles. Muscle function impairment, even mild, has dramatic effects on the quality of life. Since MuSCs yield the cellular pool required for muscle development, maintenance, and regeneration, it has been long recognized that manipulating their activity may provide new opportunities to treat muscle disorders.

Rapidly developing technologies have brought significant advances in the stem cell research field that have enabled us to better dissect the behavior of MuSCs, characterize the muscle tissue cellular diversity, and niche-mediated interactions. Yet, several decades after their discovery, the complex and exquisite regulation of MuSCs continues to fascinate researchers of the field. It is clear that MuSC activation, metabolism, and myogenesis regulation during homeostasis and in response to pathological conditions are complex processes that require fine transcriptional regulation and the orchestration of signaling pathways controlling MuSCs dynamics. A better characterization of the niche actors and their effect on MuSCs is critical for the development of novel therapies. The recent identification of myonuclei subpopulations within the muscle fibers^101–103^ is opening new avenues of research. Future studies will likely evaluate how these domains are challenged by pathological conditions and how they modulate MuSC function. Computational strategies have been recently used for predicting/modeling MuSC–niche interactions and niche-dependent cellular-conversion^104^. It is reasonable to assume that in the future bioinformatics combined with innovative technologies, such as in situ sequencing and 3D imaging will provide a deeper understanding of skeletal muscle cellular heterogeneity and dynamics in order to accelerate the race for effective treatments to alleviate muscle disorders.
